# Supplementary figures and images for: Correcting for batch effects in case-control microbiome studies
Source: PLoS Comput Biol. 2018 Apr 23;14(4):e1006102. doi: 10.1371/journal.pcbi.1006102 (PMC5940237; doi:10.1371/journal.pcbi.1006102)

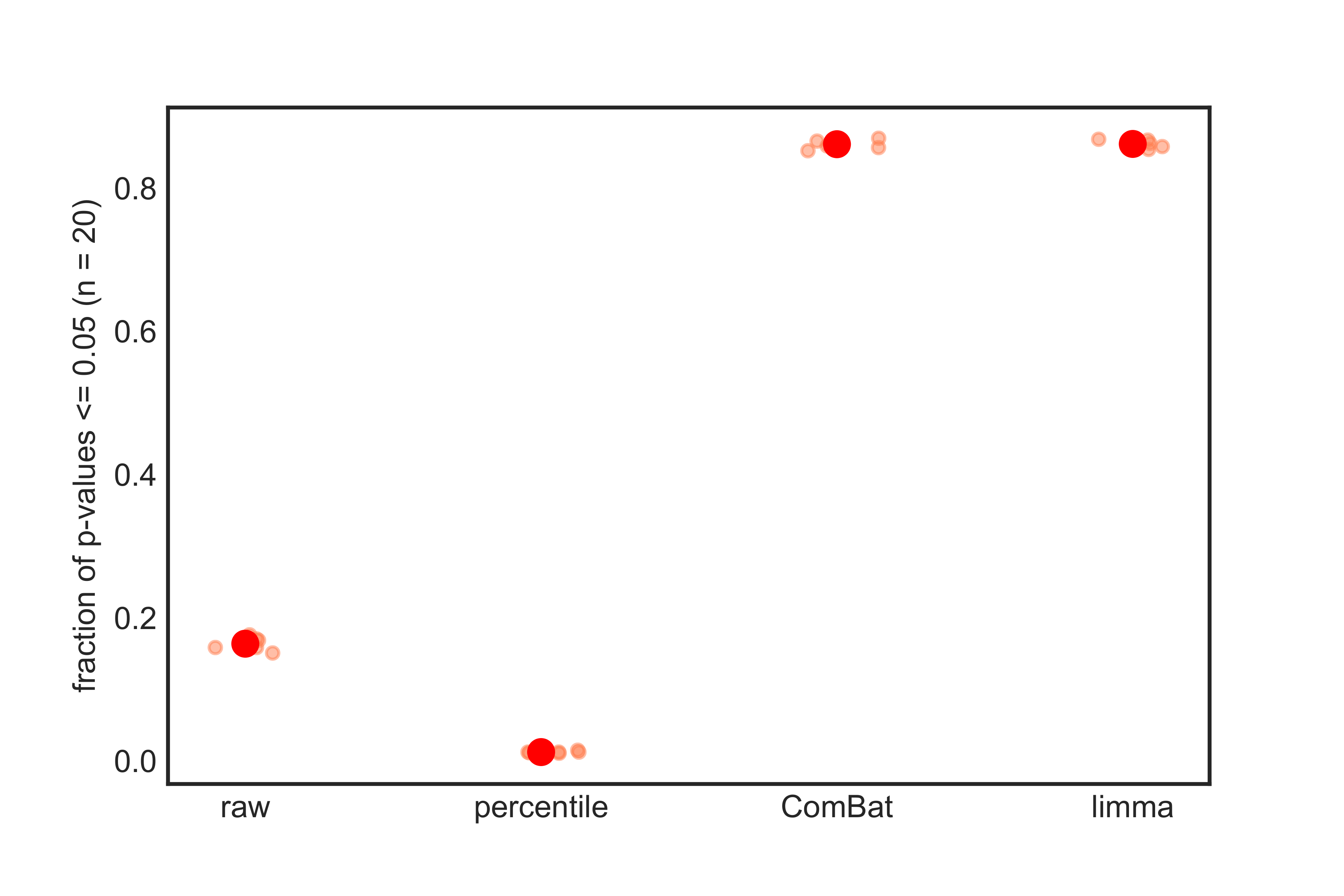

Supplement: S1 Fig — Random sets of 40 Baxter controls and random sets of 40 Zeller controls were selected for null case-control comparisons (20 iterations). Smaller points show the fraction of p-values ≤ 0.05 within a given iteration, while larger dots show the average value across all 20 iterations. Within each category, smaller points are randomly jittered along the x-axis for better visualization. The fraction of p-values ≤ 0.05 is highly inflated for all methods except percentile-normalization (red dashed line shows the null-expectation for p-values). All OTUs were included in this analysis (i.e. no abundance filter prior to running tests). ComBat and limma show highly skewed p-value distributions when including low-abundance OTUs. (TIFF) [file pcbi.1006102.s002.tiff]

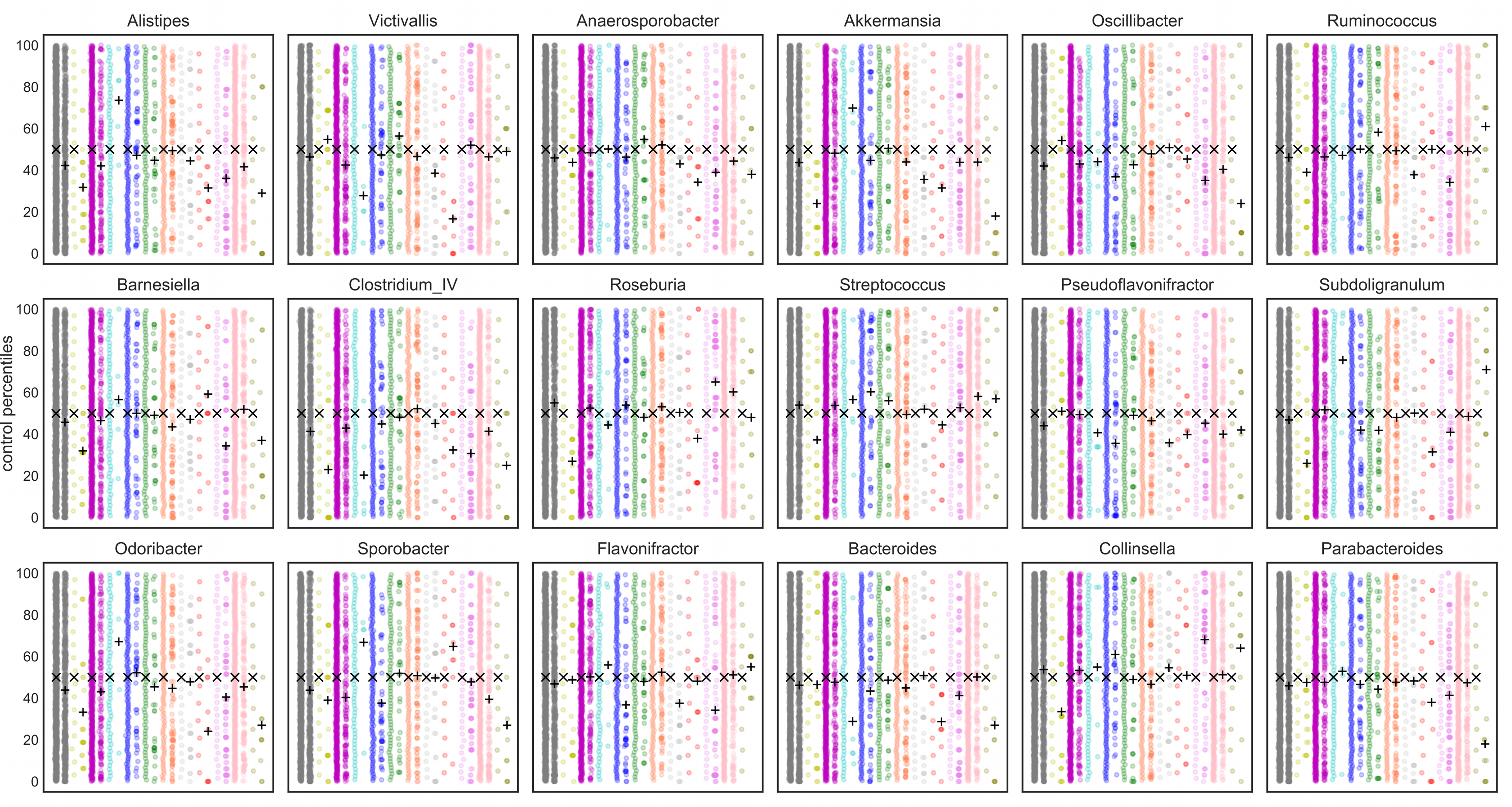

Supplement: S2 Fig — Gray points show data pooled from all 11 obesity studies. Other colors show individual obesity studies. Studies listed in order from left to right: Zhu et al. (2014), Zeevi et al. (2015), Wu et al. (2011), Baxter et al. (2016), Schubert et al. (2014), Zupancic et al. (2012), Ross et al. (2015), Jumpertz et al. (2011), Turnbaugh et al. (2009), Goodrich et al. (2014), Escobar et al. (2014). X symbols indicate the average control percentiles, while + symbols indicate the average case percentiles. (TIFF) [file pcbi.1006102.s003.tiff]

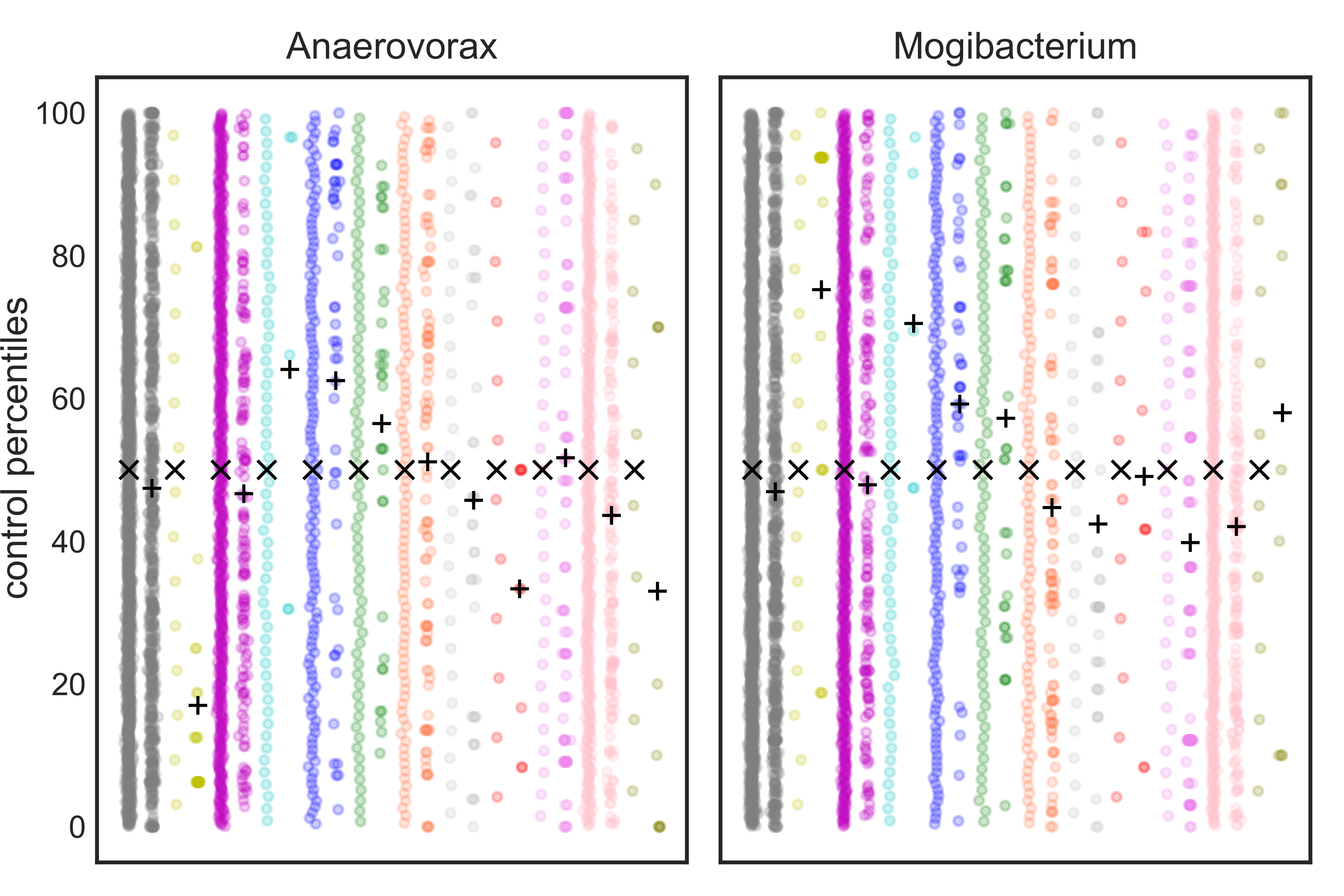

Supplement: S3 Fig — Gray points show data pooled from all 11 obesity studies. Other colors show individual obesity studies. Studies listed in order from left to right: Zhu et al. (2014), Zeevi et al. (2015), Wu et al. (2011), Baxter et al. (2016), Schubert et al. (2014), Zupancic et al. (2012), Ross et al. (2015), Jumpertz et al. (2011), Turnbaugh et al. (2009), Goodrich et al. (2014), Escobar et al. (2014). X symbols indicate the average control percentiles, while + symbols indicate the average case percentiles. (TIFF) [file pcbi.1006102.s004.tiff]
